# Shear bond strength of calcium silicate-based cements to glass ionomers

**DOI:** 10.1186/s12903-024-03890-x

**Published:** 2024-01-28

**Authors:** Ruken Ergül, Seçkin Aksu, Seçil Çalışkan, Nuray Tüloğlu

**Affiliations:** 1 Private Clinic, Eskişehir, Turkey; 2 Department of Pediatric Dentistry, Faculty of Dentistry, Mersin University, Çiftlikköy Campus, Yenişehir, Mersin, Turkey; 3 Department of Pediatric Dentistry, Faculty of Dentistry, Eskişehir Osmangazi University, Eskişehir, Turkey

**Keywords:** Adhesive, Biomaterials, Vital pulp therapy

## Abstract

**Background:**

A shear bond strength between the biomaterial and restorative material is crucial for minimizing bacterial microleakage and ensuring a favorable long-term prognosis for vital pulp therapy. This study aimed to conduct a comparative evaluation of the shear bond strength between calcium silicate-based biomaterials utilized in vital pulp treatment and various glass ionomer cement materials, both with and without the application of adhesive agents.

**Methods:**

A total of 270 acrylic blocks, each featuring cavities measuring 4 mm in diameter and 2 mm in depth, were prepared. Calcium silicate-containing biomaterials (ProRoot MTA, Medcem Pure Portland Cement, and Medcem MTA), following manufacturers’ instructions, were placed within the voids in the acrylic blocks and allowed to set for the recommended durations. The biomaterial samples were randomly categorized into three groups based on the restorative material to be applied: conventional glass ionomer cement, resin-modified glass ionomer cement, and bioactive restorative material. Using cylindrical molds with a diameter of 3.2 mm and a height of 3 mm, restorative materials were applied to the biomaterials in two different methods, contingent on whether adhesive was administered. After all samples were incubated in an oven at 37 °C for 24 h, shear bond strength values were measured utilizing a universal testing device. The obtained data were statistically evaluated using ANOVA and post-hoc Tukey tests.

**Results:**

The highest shear bond strength value was noted in the Medcem MTA + ACTIVA bioactive restorative material group with adhesive application, while the lowest shear bond strength value was observed in the ProRoot MTA White + Equia Forte HT Fil group without adhesive application (*P* < 0.05).

**Conclusion:**

Activa Bioactive Restorative may be considered a suitable restorative material in combination with calcium silicate-based biomaterials for vital pulp treatment. The application of adhesives to calcium silicate-based biomaterials can effectively address the technical limitations.

## Background

The primary purpose of pulp treatment is to safeguard the structural integrity of dental tissues while preserving the vitality of the pulp, which may be compromised due to caries, traumatic injury, or other factors. The dentin-pulp complex possesses inherent physiological defense mechanisms, such as reparative dentinogenesis, which play a crucial role in maintaining pulp vitality [1]. Consequently, in contemporary dental practice, preserving pulp vitality through biologically based approaches holds significant importance, with vital pulp therapy (VPT) emerging as the principal treatment modality for conserving healthy pulp tissue in cases of deep dentin caries and pulp exposure resulting from caries [2]. The success of VPT relies on the application of materials and establishment of a hermetic seal. Furthermore, strong shear bond strength (SBS) between the biomaterial and restorative material is imperative to minimize bacterial microleakage and ensure a favorable long-term prognosis for VPT [3].

Several studies have delved into the efficacy of Proroot MTA in comparison to various calcium silicate-based materials like Biodentine. Conclusions drawn from these investigations indicate that while Proroot MTA exhibits significantly enhanced clinical performance, it may trail behind more contemporary materials in certain aspects [4, 5]. There is currently only one study on the SBS of Medcem MTA and Medcem Pure Portland Cement, which are prepared to meet all MTA indicators accepted in the market and are claimed by the manufacturer to exhibit extraordinary mechanical properties [6]. Hence, it is of utmost importance to compare them with well-established material in clinical practice for an extended duration, such as Proroot MTA. Moreover, considering that the procedure steps should be kept as short as possible in pediatric dentistry, more research is needed to increase the current research as well as specific comparisons to current materials to understand how important the effect of adhesive application is on the SBS between biomaterial and restorative material. Therefore, the objective of this study is to compare the SBS of calcium silicate-based biomaterials utilized in VPT with the SBS of glass ionomer restorative materials. Additionally, the study aims to assess the impact of adhesive application on SBS.

## Materials and methods

The present study utilized three calcium silicate-based biomaterials, WMTA, MMTA, and MPPC, along with three glass ionomer restorative materials, Equia Forte HT Fil (EFHF), Fuji II LC (FIILC), and Activa Bioactive-Restorative (ABR). Additionally, a self-etch adhesive material, Clearfil Se Bond, was used (Table 1).

**Table 1:** The materials used in the study

Material	Chemical composition	Lot Number	Manufacturer
ProRoot MTA White (WMTA)	Tricalcium-silicate, Dicalcium-silicate, Tricalcium aluminate, Calcium sulfate dihydrate, Bismuth oxide	0000266733	Dentsply, Tulsa Dental, OK, USA
Medcem MTA (MMTA)	Tricalcium-silicate, Tricalcium aluminate, Dicalcium-silicate, Tetracalcium aluminoferrite	MTZ181020	Weinfelden, Switzerland
Medcem Pure Portland Cement (MPPC)	Tricalcium-silicate, Tricalcium aluminate, Dicalcium-silicate, Tetracalcium Alumino Ferrite, Zirconium oxide	RX181020	Weinfelden, Switzerland
Equia Forte HT Fil (EFHF)	Powder: Fluoroalumina silicate glass Liquid: Polyacrylic acid	2,101,291	GC Corp, Tokyo, Japan
Fuji II LC (FIILC)	Fluoroaluminosilicate glass, HEMA, Polybasic carboxylic acid, Urethane dimethacrylate, Camphoroquinone, Distilled water	2,101,061	GC Corp, Tokyo, Japan
Activa Bioactive-Restorative (ABR)	Modified mixture of diurethane and other methacrylates Polyacrylic acid, Amorphous silica, Sodium fluoride	201,229	Pulpdent Corp, Watertown, USA
Clearfil SE Bond	Primer: 10-MDP, HEMA, Hydrophilic dimethacrylate, Photo-initiator, Water Adhesive: 10-MDP, HEMA, Bis-GMA, Hydrophilic dimethacrylate, Dl-camphoroquinone, Silanated colloidal Silica, Initiator	000134	Kuraray Noritake, Japan

This research was conducted at the Eskişehir Osmangazi University Research Laboratory. A total of 270 acrylic blocks, each featuring cylindrical cavities with a diameter of 4 mm and depth of 2 mm, were prepared for the study. The sample size was determined based on an effect size of 0.25, with a statistical power of 94.8%, and significance level of 5% [7]. Each biomaterial consisted of 90 specimens, and the calcium silicate-based materials were carefully placed within the cavities of the acrylic blocks following the manufacturer’s instructions.

The surfaces of the calcium silicate-based material samples were carefully covered with moist cotton pellets and a temporary filling material (Cavit, 3 M ESPE, USA). Subsequently, the samples were placed in an oven set at 100% humidity and maintained at 37 °C using a Nüve ES 252 incubator (Nüve Sanayi Malzemeleri Manufacturing and Trading İnc., Ankara, Turkey) for 4 h to allow complete hardening of the materials. After removing the samples from the oven, the temporary filling material covering the biomaterials was removed gently. Surface polishing of the biomaterials was performed using an aluminum oxide disc (Tor; Tor VM Ltd., Moscow, Russia).

The prepared biomaterial samples were then randomly allocated to three groups, with each group consisting of 15 samples corresponding to a specific glass ionomer restorative material. Subsequently, the application of the glass ionomer restorative material was initiated. Following the application of the light-cured glass ionomer materials, polymerization was accomplished using an LED light device (Elipar Deep Cure-L, 3 M Espe Corporation, CA, USA; light power:1100 mW/cm²) (LOT NO:6,500,263).

The glass ionomer restorative materials were applied to cylindrical plastic molds measuring 3.2 mm in diameter and 3 mm in height, which were placed on acrylic blocks along with the calcium-silicate samples, following the manufacturer’s instructions. The application of glass ionomer restorative materials to biomaterials was carried out using two distinct methods. In the first method, glass ionomer restorative materials are directly bonded to biomaterials without the application of an adhesive. In the second method, the glass ionomer restorative materials were bonded to the biomaterials by applying an adhesive (Clearfil SE Bond, Kuraray, Noritake, Japan), according to the manufacturer’s instructions.

**Fig. 1:**
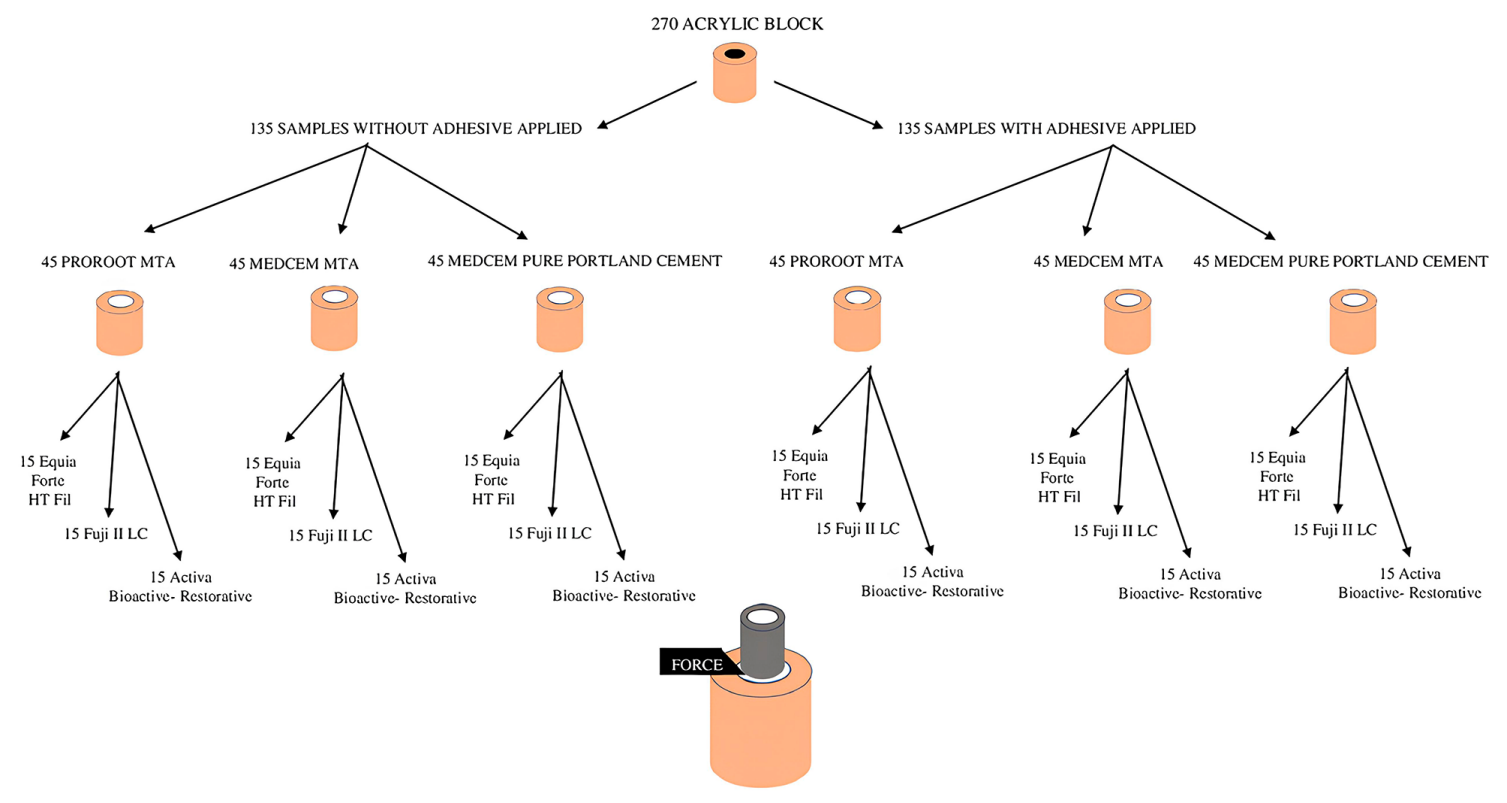
Flowchart of experimental procedure

Following a 24-hour incubation period at 37 °C and 100% humidity, the samples were placed in a universal testing machine (MOD Dental MIC-101, Esetron Smart Robotechnologies, Ankara, Turkey) to measure adhesive strength. The testing device was configured to operate at a constant speed of 1 mm/min, and a force parallel to the long axis of the adhesion area was applied until the fracture of the bond occurred. The resulting force, measured in Newtons, was divided by the surface area to calculate the adhesive strength, which was recorded in megapascals (MPa) by a single observer. A flowchart of the experimental procedure is represented in Fig. 1.

Scanning Electron Microscopy (SEM) was used to analyze the fracture patterns. All experimental groups were carefully selected and placed in labeled containers, followed by dehydration using a series of ethanol solutions of varying concentrations. Subsequently, the samples were dried in a vacuum oven at 60 Â °C. The dried samples were then examined under a Hitachi Regulus 8230 field-emission scanning electron microscope (Tokyo, Japan) at a magnification of x1000. Representative areas of interest were identified and photographed to document the observed features.

Data analysis was performed using Microsoft Excel 2010 (Microsoft Corporation, USA) and SPSS 25.0 (IBM, Chicago, USA). Statistical evaluation of the data was conducted using one-way analysis of variance (ANOVA). In cases where a significant difference was observed, a post-hoc Tukey test was employed to determine specific group differences. Statistical significance was defined as *P* < 0.05.

## Results

Upon evaluating the SBS of WMTA and glass ionomer-containing restorative materials, the ABR group demonstrated the highest average SBS value (*P* < 0.05), exhibiting a statistically significant difference between the EFHF and FIILC groups. Notably, no statistically significant difference was found (*P* > 0.05). When the SBS of MMTA and glass ionomer-containing restorative materials, the EFHF group displayed the highest average SBS value (*P* < 0.05). Moreover, it was determined that there was no statistically significant difference between the FIILC and ABR groups (*P* > 0.05). While evaluating the SBS of MPPC and glass ionomer-containing restorative materials, it was determined that there was no statistically significant difference between the groups (*P* > 0.05). The FIILC group exhibited the highest average SBS value (10.29 ± 11.64 MPa), while the EFHF group displayed the lowest average SBS value (6.36 ± 4.95 MPa).

Among the samples in the groups without adhesive application, the MMTA + EFHF group exhibited the highest average SBS value (21.48 ± 8.95 MPa), while the WMTA + EFHF group displayed the lowest average SBS value (4.94 ± 2.13 MPa). The WMTA + FIILC group exhibited the lowest average SBS value among all samples with adhesive application. Conversely, the ABR group demonstrated the highest average SBS value among all biomaterial samples applied with adhesive. The identification of significant differences in the statistical comparison between non-adhesive groups and adhesive groups for the same biomaterial-restorative material combination can be found in Tables 2, 3 and 4.

**Table 2:** Average SBS values of ProRoot MTA White samples with and without adhesive application to restorative materials

WMTA	Adhesive Applied Group	Not Adhesive Applied Group	*P*
**EFHF**	12.99 ± 7.57	4.94 ± 2.13	0.01*
**FIILC**	7.63 ± 3.29	7.41 ± 2.95	0.848
**ABR**	22.54 ± 14.15	14.47 ± 5.37	0.054

One-way ANOVA –Tukey Test. SBS: Shear Bond Strength, WMTA: ProRoot MTA White, EFHF: Equia Forte HT Fil, FIILC: Fuji II LC, ABR: Activa Bioactive-Restorative. Statistical significance level was accepted as *P* < 0.05

**Table 3:** Average SBS values of Medcem MTA samples with and without adhesive application to restorative materials

MMTA	Adhesive Applied Group	Not Adhesive Applied Group	*P*
**EFHF**	12.91 ± 10.20	21.48 ± 8.9	0.021*
**FIILC**	8.90 ± 3.88	9.79 ± 2.83	0.480
**ABR**	30.57 ± 16.16	12.0 ± 4.50	0.01*

One-way ANOVA –Tukey Test. SBS: Shear Bond Strength, MMTA: Medcem MTA, EFHF: Equia Forte HT Fil, FIILC: Fuji II LC, ABR: Activa Bioactive-Restorative. Statistical significance level was accepted as *P* < 0.05.

**Table 4:** Average SBS values of Medcem Pure Portland Cement samples with and without adhesive application to restorative materials

MPPC	Adhesive Applied Group	Not Adhesive Applied Group	*P*
**EFHF**	9.83 ± 3.95	6.36 ± 4.95	0.043*
**FIILC**	7.25 ± 4.83	10.29 ± 11.64	0.358
**ABR**	18.59 ± 7.40	9.85 ± 4.62	0.01*

One-way ANOVA –Tukey Test. SBS: Shear Bond Strength, MPPC: Medcem Pure Portland Cement, EFHF: Equia Forte HT Fil, FIILC: Fuji II LC, ABR: Activa Bioactive-Restorative. Statistical significance level was accepted as *P* < 0.05.

**Fig. 2:**
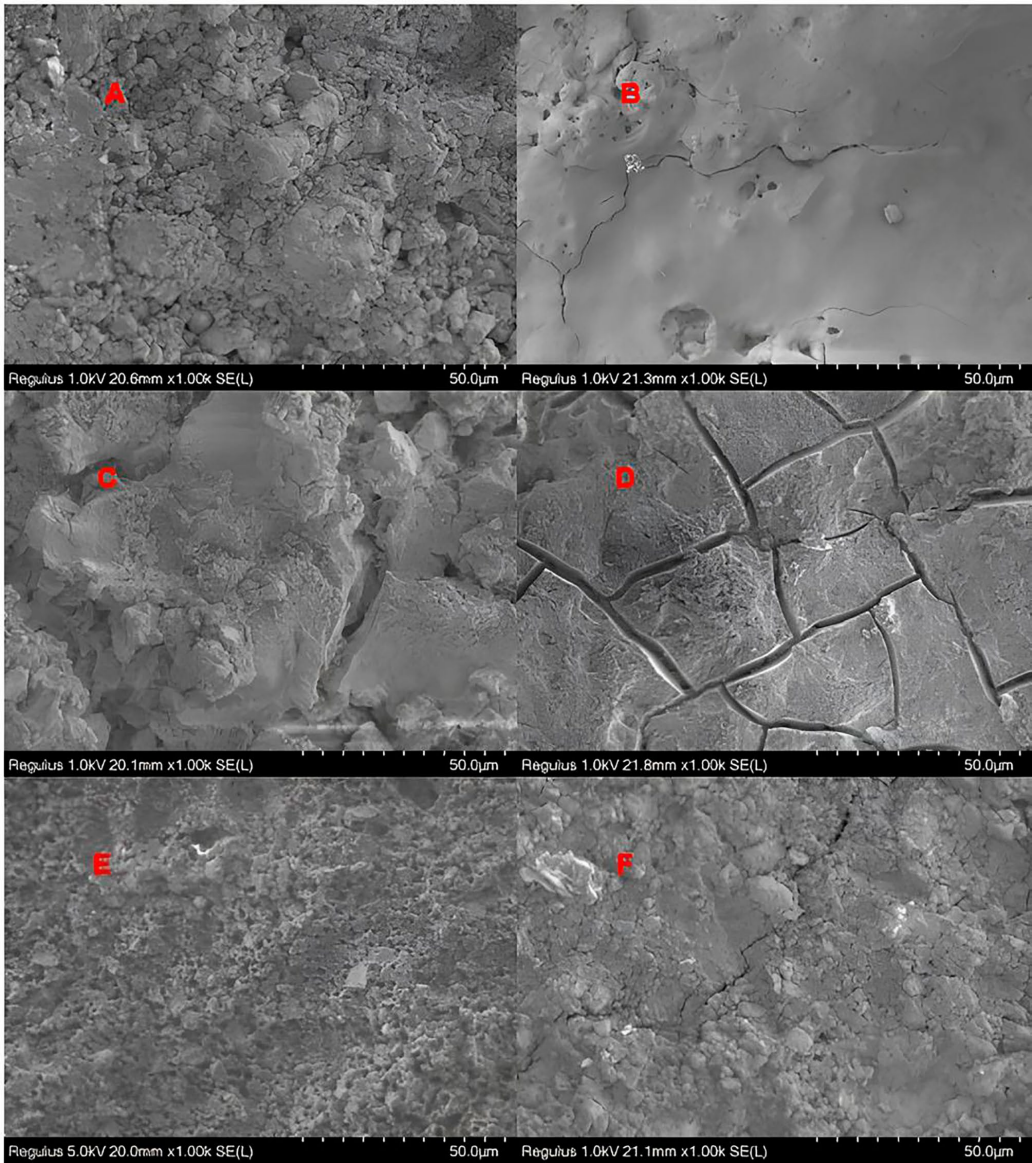
SEM micrographs of the ProRoot MTA White at 1000x magnification. (**A**) ProRoot MTA White- Equia Forte HT Fil (non-adhesive group): Mix Fracture. (**B**) ProRoot MTA White- Equia Forte HT Fil (adhesive applied group): Cohesive Fracture in ProRoot MTA White. (**C**) ProRoot MTA White- Fuji II LC (non-adhesive group): Cohesive Fracture in ProRoot MTA. (**D**) ProRoot MTA White- Fuji II LC (adhesive applied group): Adhesive Fracture. (**E**) ProRoot MTA- Activa Bioactive-Restorative (non-adhesive): Mix Fracture. (**F**) ProRoot MTA- Activa Bioactive-Restorative (adhesive applied group): Mix Fracture

**Fig. 3:**
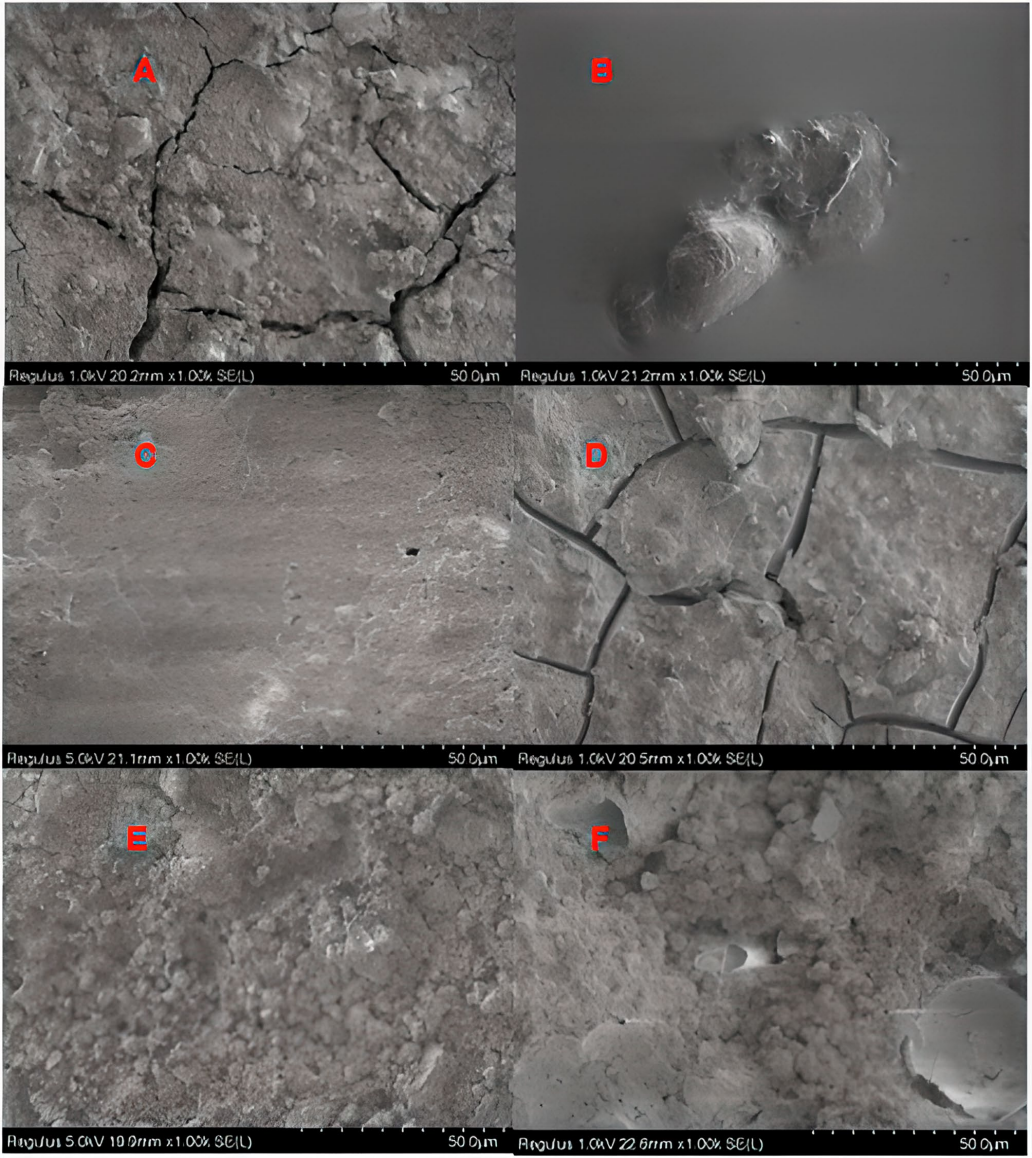
SEM micrographs of the Medcem MTA at 1000x magnification. (**A**) Medcem MTA- Equia Forte HT Fil (non-adhesive group): MMTA- Cohesive Fracture. (**B**) Medcem MTA- Equia Forte HT Fil (adhesive applied group): Cohesive Fracture in Medcem MTA. (**C**) Medcem MTA- Fuji II LC (non-adhesive group): Cohesive Fracture in Fuji II LC. (**D**) Medcem MTA-FIILC (adhesive applied group): Mix Fracture. (**E**) Medcem MTA- Activa Bioactive-Restorative (non-adhesive group): Cohesive Fracture in Medcem MTA (**F**) Medcem MTA- Activa Bioactive-Restorative (adhesive applied group): Cohesive Fracture in Medcem MTA

**Fig. 4:**
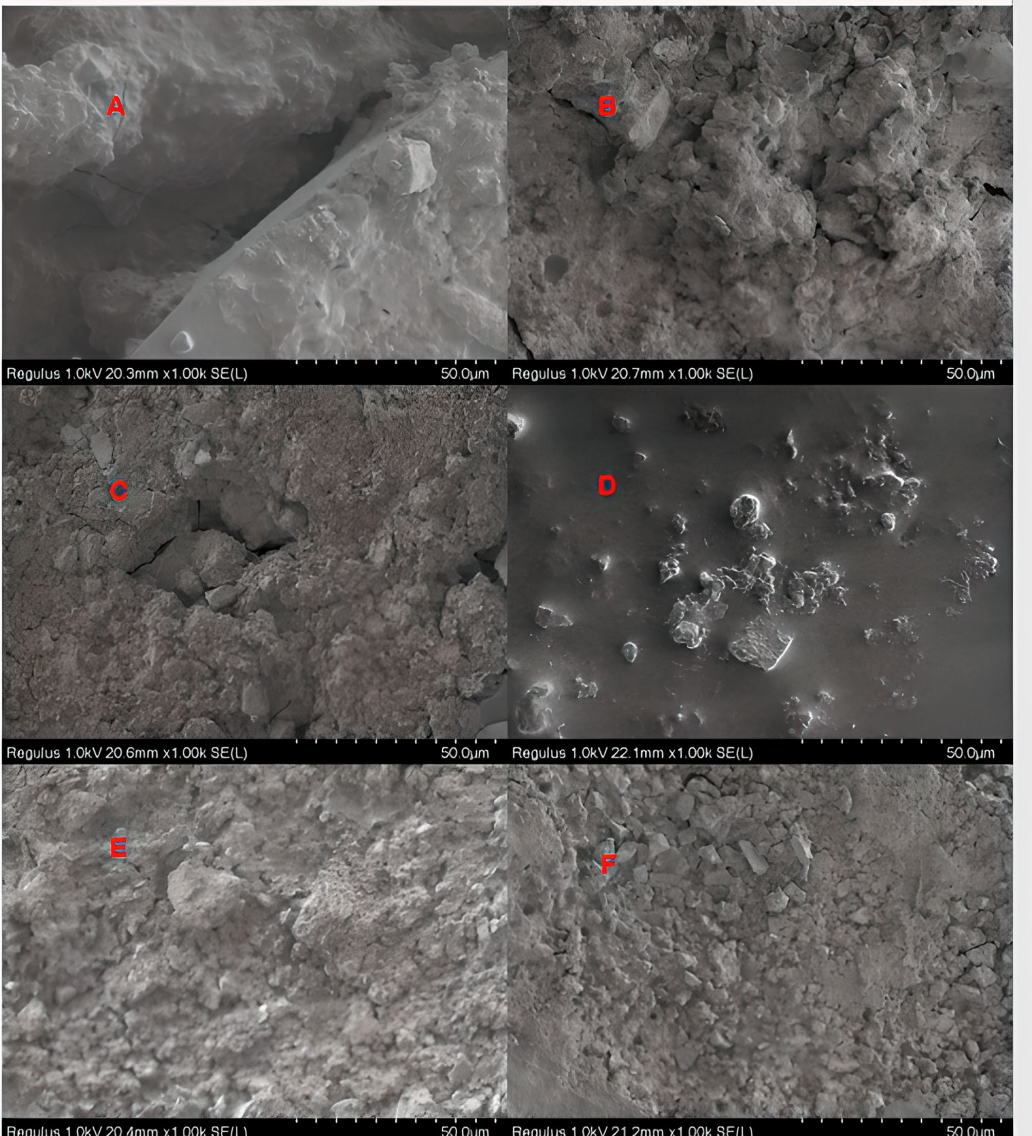
SEM micrographs of the Medcem Pure Portland Cement at 1000x magnification. (**A**) Medcem Pure Portland Cement- Equia Forte HT Fil (non-adhesive group): Mix Fracture. (**B**) Medcem Pure Portland Cement- Equia Forte HT Fil (adhesive applied group): Mix Fracture. (**C**) Medcem Pure Portland Cement- Fuji II LC (non-adhesive group): Cohesive Fracture in Medcem Pure Portland Cement. (**D**) Medcem Pure Portland Cement- Fuji II LC (adhesive applied group): Adhesive Fracture. (**E**) Medcem Pure Portland Cement- Activa Bioactive-Restorative (non-adhesive group): Mix Fracture. (**F**) Medcem Pure Portland Cement- Activa Bioactive-Restorative (adhesive applied group): Mix Fracture

Upon examination of fracture types between biomaterial and restorative material, cohesive failure was found to be the most common in all groups (*P* < 0.05). Cohesive fractures were exclusively observed in biomaterials. The MMTA group exhibited the highest fracture frequency, while the MPPC group displayed the lowest fracture frequency (Table 5). Although mix fractures were most common in the MPPC group, this difference was not statistically significant (*P* > 0.05) Examples of the most common fractural patterns in SEM micrographs of materials at 1000x magnification are presented in Figs. 2, 3 and 4.

**Table 5:** Fracture type percentages in Biomaterial-Restorative materials

	Adhesive Fracture		Cohesive Fracture		Mix Fracture	
	N	%	N	%	N	%
WMTA	21	23.33	58	64.44	11	12.22
MMTA	19	21.11	66	73.33	5	5.55
MPPC	36	40.00	41	45.56	13	14.44

## Discussion

Vital pulp treatment is frequently used by pediatric dentists to eradicate bacteria within the dentin-pulp complex, preserve pulp vitality, and create a conducive environment for apexogenesis [8]. Successful VPT necessitates stable pulpal hemodynamics and the establishment of a hermetic coronal restoration to eliminate severe inflammatory reactions [9]. The ideal biomaterial utilized in VPT should promote dentin formation by stimulating residual pulp tissue and possess the ability to resist long-term bacterial infiltration when restorative materials are applied [10]. Hence, the bonding between restorative materials and biomaterials is of utmost importance. Failure to achieve hermetic occlusion at the interface between the biomaterial and restorative material would result in bacterial penetration into the pulp, ultimately compromising the success of the VPT procedure [11].

Biomaterials and restorative materials must possess favorable compressive strength to withstand masticatory forces [12]. To minimize microleakage beneath composite resin restorations, the use of glass ionomer cement or resin-modified glass ionomer cement as bases has frequently been suggested [13]. A review of the literature pertaining to the SBS of calcium silicate-based materials revealed that the majority of studies have focused on WMTA [4, 14–17], with limited investigations conducted on MMTA and MPPC [18]. The objective of our research was to compare the SBS of WMTA, MMTA, and MPPC-calcium silicate-based biomaterials commonly employed in VPT with those of various restorative materials (EFHF, FIILC, and ABR).

One of the prevalent in vitro approaches employed to assess the adhesive characteristics of restorative materials is the examination of bond strength [19]. Adhesive assessments encompass both quantitative analyses and qualitative screening tests, which aid in predicting the load-bearing capacity and longevity of the bond while also facilitating the investigation of adhesive interfaces and adhesion failures [20]. MTA exhibits brittleness, rendering it unsuitable for tensile bond strength testing [21]. Consequently, in this investigation, the SBS test was employed to evaluate the bond strength of calcium silicate-based biomaterials with various restorative materials containing glass ionomers.

In this research, the group with the lowest average SBS value for all samples, without adhesive application, was observed in the WMTA + EFHF group. Likewise, Bicer et al. [22] observed that WMTA samples lacking any binding agent exhibited a comparatively lower SBS value (5 ± 0.50 MPa) to EFHF when compared to resin-modified glass ionomer cement and compomer. Similarly, in an investigation conducted by Cantekin and Avcı [4], the glass ionomer cement group had the lowest SBS within the WMTA group, where no adhesive was applied.

Tulumbacı et al. [23] reported a mean SBS of 2.84 ± 3.51 MPa in the resin modified glass ionomer cement (RMGIC) group (utilizing Photac Fil Quick Applicap) when combined with WMTA without adhesive. Interestingly, in this study, it was found that the mean SBS between WMTA without adhesive and FIILC was higher (7.41 ± 2.95 MPa). In this study, the average SBS value of the non-adhesive version of the WMTA + FIILC combination was determined to be statistically significantly lower than the version where the adhesive was applied. It is hypothesized that this disparity may be attributed to variations in the composition and particle size of different brands of glass ionomer cement. In a study conducted by Bicer et al. [22], which runs parallel to the aforementioned investigation, the mean SBS between WMTA without adhesive and FIILC was reported as 6.22 ± 0.84 MPa.

As ABR is a novel material, no existing literature has been identified that evaluated the SBS in relation to established biomaterials. Our study, however, revealed that ABR demonstrated significantly higher SBS values in various groups, except for the MPPC group without adhesive. ABR incorporates an ionic resin component containing phosphate acid groups [24]. We hypothesize that, through a water-dependent ionization process, the hydrogen ions dissociate from the phosphate groups and are substituted by calcium ions generated from the hydration of MTA. This phenomenon consequently enhances the strength of the bond.

In this research, the group with the lowest average SBS value for all samples without adhesive application was observed in the MMTA + EFHF group. Additionally, the lowest average SBS value for all adhesive-applied samples was detected in the MMTA + FIILC group. However, in this study, the mean SBS of the adhesive-free MMTA and EFHF group (21.48 ± 8.95 MPa) showed a significant increase compared to the values reported by Duman et al. (5.76 ± 3.63 MPa). Similarly, the mean SBS of the adhesive-free MMTA and FIILC group (9.79 ± 2.83 MPa) also exhibited higher values than those reported in the study conducted by Duman et al. (6.06 ± 5.75 MPa) [6]. The observed difference in the mean SBS between the glass ionomer cement and MMTA groups in this study may be attributed to various factors, including variations in research conditions, sample size, and differences among practitioners.

Duman et al. [6] reported the SBS value of 37.27 ± 18.81 MPa for the EFHF group with MPPC without adhesive, whereas our study yielded a value of 6.36 ± 4.95 MPa. It is important to note that the stress distribution in shear bond tests can be intricate, leading to variations in results among different researchers. The heterogeneity observed in these outcomes may also be attributed to several bonding variables, including sample storage conditions, sample characteristics, surface preparation techniques, thermal cycling, and film thickness. Furthermore, variations in the results may arise owing to the diverse physical properties of calcium silicate-based biomaterials, variations in radioactive components, discrepancies in the production process, purity of the components, and differences in hydration products [6, 25]. Although the primary constituent of the biomaterials utilized in our research is calcium silicate, the recently developed MMTA incorporates zirconium oxide instead of bismuth oxide, unlike WMTA. MPPC primarily comprises dicalcium and tricalcium silicates [6, 26]. The variability observed in the study results can be attributed to the aforementioned factors, which is consistent with the results of previous investigations [6, 25]. Moreover, the mean SBS of the MPPC and FIILC groups in the non-adhesive group (10.29 ± 11.64 MPa) was similar to that reported by Duman et al. (9.80 ± 5.33 MPa) [6].

Previous studies have explored the SBS of biomaterials and restorative materials bonded without the use of adhesive agents. In the second part of our research, we extended the investigation to include the application of adhesive agents in the bonding of biomaterials and glass ionomer cement to make a valuable contribution to the existing literature [6, 11, 22]. Irrespective of the adhesive application, it was observed that the WMTA biomaterial exhibited the highest average SBS (22.54 + 14.15 MPa) when applied in conjunction with the adhesive agent. The EFHF group (12.99 + 7.57 MPa) demonstrated the next highest SBS values within the adhesive group. Upon evaluating the bond strength of restorative materials with MMTA following the application of adhesive agents, it was observed that ABR exhibited significantly higher bond values than the other materials, similar to the group without adhesive application. Additionally, for EFHF and FIILC, the groups without adhesive treatment demonstrated superior performance compared with the adhesive-applied groups. This phenomenon may be attributed to the fact that both the micromechanical and chemical bonds contribute to the adhesion between these types of cement. Previous studies have reported that the high SBS of the EFHF group without adhesive application to MTA can be attributed to two factors. First, the surface of MTA contains metallic oxides that facilitate strong chemical bonding with glass ionomer cement through metallic bonds. Second, the presence of pores on the MTA surface increases the surface area for micromechanical bonding between MTA and the glass ionomer cement [21, 27].

When the SBS of all restorative material groups was evaluated with MPPC, it was found that the highest values were reached in the ABR group in which the adhesive was applied, similar to the findings with MMTA. However, unlike MMTA, the mean SBS after the adhesive application of EFHF was higher than that in the group without adhesive application. It was observed that adhesive application did not reduce or change the bond strength value in the bonding of FIILC with all three biomaterials. The lower bond strength observed between FIILC and the biomaterials can be attributed to the bonding mechanism of this material. Mitra et al. [28] also reported that stress resulting from polymerization shrinkage and dimensional changes can compromise the adhesion of RMGICs, which supports the findings of our study. In this study, lower SBS values were observed in all adhesive-applied biomaterial-RMGIC groups than in other restorative materials containing glass ionomers. Similarly, Ajami et al. [11] reported low bond strengths between RMGIC and pulp-capping agents. We believe that the weaker bond strength of RMGIC to calcium silicate-based materials may be attributed to polymerization shrinkage caused by monomers such adps hydroxyethyml metacrylate and urethane dimethacrylate present in the cement. The application of adhesive increased the average SBS value in all biomaterial-glass ionomer-containing restorative material groups, except for the FIILC and EFHF-MMTA groups. The higher SBS in the adhesive-applied groups may be attributed to the adhesive system utilized in this study, specifically the use of Clearfil SE Bond containing a 10-MDP functional monomer. In addition to micromechanical bonding, this monomer facilitates chemical adhesion through chemical binding with calcium in biomaterials [22, 29].

Based on a comprehensive analysis of the data obtained in this study, it can be concluded that the application of ABR with adhesive as a restorative material on biomaterials effectively enhanced the bond strength. Similar to our findings, François et al. [30] demonstrated that ABR applied with a bonding agent exhibited higher shear bond values than ABR without a bonding agent. The presence of methacrylate monomers in ABR, along with its compositional similarity to composite resins, may account for the observed high shear bond strength. Following the polymerization of the resin-containing materials, the presence of unpolymerized residual monomers leads to the formation of an oxygen inhibition layer on the surface. This layer, containing a greater number of unsaturated carbon double bonds available for chemical bonding, has been reported to enhance bonding by forming strong covalent bonds with the bonding system [31]. This explanation offers insight into the observed increase in the ABR bond strength after the application of the adhesive in this study.

A high bond strength between restorative materials and biomaterials is essential to minimize microleakage. It is generally considered that a bond strength ranging from to 17–20 MPa is required to achieve well-sealed restorations and sufficient resistance to contraction forces [32]. Based on the findings of this study, only the MMTA-EFHF group exhibited values higher than 17 MPa in the non-adhesive group. In the adhesive group, the ABR + MMTA, ABR + MPPC, and ABR + WMTA groups demonstrated bond strengths exceeding 17 MPa within the ABR group.

In this study, restorative materials were applied onto the surfaces of biomaterials after 4 h, according to the manufacturer’s recommendations. However, it is worth noting that in clinical settings, restorative material applications of calcium silicate-based biomaterials are typically performed after a minimum of 24 h. This disparity in timing may limit the generalizability of our findings to other clinical applications. It is important to recognize that while MTA undergoes initial setting within a short timeframe, it is recommended to delay the placement of permanent restorations for at least 72 h or longer. This allows for improved resistance to dislodgement, enhanced sealing, and optimal physical properties [33]. The results obtained in this study can serve as a valuable reference for the design of future in vivo and in vitro studies to address this limitation. Considering the influence of bonding variables, it is advisable to incorporate dentin and enamel surface properties as additional guiding factors in future investigations.

The null hypotheses formulated for this study are as follows: there is no statistically significant difference in the SBS between the tested biomaterials and restorative materials, and the adhesive application does not exert a statistically significant effect on the bond strength between the biomaterial and restorative material. Based on the findings of this study, it can be concluded that the SBS values of the biomaterial-glass ionomer-containing restorative material groups exhibited significant variability, thus rejecting the null hypotheses. However, it is important to note that in vitro studies cannot fully replicate all clinical aspects or accurately predict clinical behavior. Further prospective clinical studies are warranted to validate these findings. Additionally, there is a need for future studies to analyze the interfaces between new biomaterials and restorative cements in the presence of saliva contamination as well as to consider the completion of the setting reactions of calcium silicate-based biomaterials. These areas of investigation will contribute to a more comprehensive understanding of the behavior and performance of these materials in clinical scenarios.

## Conclusion

Activa Bioactive Material, a contemporary substance incorporating calcium silicate, demonstrates elevated shear strength, suggesting its suitability for vital pulp treatment in pediatric dentistry. Furthermore, the application of adhesive proves effective in surmounting the technical challenges associated with the immediate placement of restorative materials on calcium silicate-based biomaterials following the initial setting.

## Data Availability

The data that support the findings of this study are available from the corresponding author upon reasonable request.

## Abbreviations

VPT: Vital pulp therapy
SBS: Shear bond strength
WMTA: ProRoot MTA White
MMTA: Medcem MTA
MPPC: Medcem Pure Portland Cement
EFHF: Equia Forte HT Fil
FIILC: Fuji II LC
ABR: Activa Bioactive-Restorative
SEM: Scanning Electron Microscopy
RMGIC: Resin modified glass ionomer cement

## References

[1] Rodriguez-Lozano FJ, Lopez-Garcia S, Garcia-Bernal D, Sanz JL, Lozano A, Pecci-Lloret MP (2021). Cytocompatibility and bioactive properties of the new dual-curing resin-modified calcium silicate-based material for vital pulp therapy. Clin Oral Investig.

[2] Dingming H, Qian L, Qian L, Ling Y, Xuedong Z (2017). Confusion and solution for vital pulp therapy. Hua Xi Kou Qiang Yi Xue Za Zhi.

[3] Akhlaghi N, Khademi A (2015). Outcomes of vital pulp therapy in permanent teeth with different medicaments based on review of the literature. Dent Res J (Isfahan).

[4] Cantekin K, Avci S (2014). Evaluation of shear bond strength of two resin-based composites and glass ionomer cement to pure tricalcium silicate-based cement (Biodentine(R)). J Appl Oral Sci.

[5] Kaup M, Dammann CH, Shafer E, Dammaschke T (2015). Shear bond strength of Biodentine, ProRoot MTA, glass ionomer cement and composite resin on human dentine ex vivo. Head Face Med.

[6] Duman S, Çalışkan A, Çalışkan S (2021). Comparison of Medcem MTA, Medcem Pure Portland Cement and NeoMTA to Pediatric Restorative materials to Shear Bond Strength. NEUDentJ.

[7] Palma PJ, Marques JA, Falacho RI, Vinagre A, Santos JM, Ramos JC (2018). Does delayed Restoration Improve Shear Bond Strength of different restorative protocols to Calcium Silicate-based cements?. Mater (Basel).

[8] Duncan HF (2022). Present status and future directions-vital pulp treatment and pulp preservation strategies. Int Endod J.

[9] Urkande NK, Mankar N, Nikhade PP, Chandak M (2023). Beyond Tradition: non-surgical endodontics and Vital Pulp Therapy as a dynamic combination. Cureus.

[10] Schmidt A, Schafer E, Dammaschke T (2017). Shear Bond Strength of Lining Materials to calcium-silicate cements at different time intervals. J Adhes Dent.

[11] Ajami AA, Navimipour EJ, Oskoee SS, Kahnamoui MA, Lotfi M, Daneshpooy M (2013). Comparison of shear bond strength of resin-modified glass ionomer and composite resin to three pulp capping agents. J Dent Res Dent Clin Dent Prospects.

[12] Al-Sherbiny IM, Farid MH, Abu-Seida AM, Motawea I, Bastawy HA (2021). Chemico-physical and mechanical evaluation of three calcium silicate-based pulp capping materials. Saudi Dent J.

[13] Arandi NZ, Rabi T (2020). Cavity bases revisited. Clin Cosmet Investig Dent.

[14] Alzraikat H, Taha NA, Qasrawi D, Burrow MF (2016). Shear bond strength of a novel light cured calcium silicate based-cement to resin composite using different adhesive systems. Dent Mater J.

[15] Bayrak S, Tunc ES, Saroglu I, Egilmez T (2009). Shear bond strengths of different adhesive systems to white mineral trioxide aggregate. Dent Mater J.

[16] Doozaneh M, Koohpeima F, Firouzmandi M, Abbassiyan F (2017). Shear Bond Strength of Self-Adhering Flowable Composite and Resin-modified Glass Ionomer to two pulp capping materials. Iran Endod J.

[17] Shin JH, Jank JH, Park SH, Kim E (2014). Effect of mineral trioxide aggregate surface treatments on morphology and bond strength to composite resin. J Endod.

[18] Sismanoglu S, Yildirim-Bilmez Z, Gurcan AT, Gumustas B, Taysi M, Berkman M (2022). Influence of intracoronal bleaching agents on the bond strength of MTA cements to composite resin and their surface morphology. Odontology.

[19] Tunc ES, Saroglu-Sonmez I, Bayrak S, Egilmez T (2008). The evaluation of bond strength of a composite and a compomer to white mineral trioxide aggregate with two different bonding systems. J Endod.

[20] Xavier MT, Costa AL, Caramelo FJ, Palma PJ, Ramos JC. Evaluation of the interfaces between restorative and regenerative biomaterials used in vital pulp therapy. Mater (Basel). 2021;14(17). 10.3390/ma14175055. 10.3390/ma14175055 PMC8434275 34501145

[21] Ajami AA, Bahari M, Hassanpour-Kashani A, Abed-Kahnamoui M, Savadi-Oskoee A, Azadi-Oskoee F (2017). Shear bond strengths of composite resin and giomer to mineral trioxide aggregate at different time intervals. J Clin Exp Dent.

[22] Bicer H, Bayrak S (2019). Vital pulpa tedavisinde kullanılan kalsiyum silikat içerikli biyomateryallerin restoratif materyallere bağlanma dayanımının değerlendirilmesi. Selçuk Dent J.

[23] Tulumbaci F, Erkmen Almaz F, Arikan V, Mutluay MS (2017). Shear bond strength of different restorative materials to mineral trioxide aggregate and Biodentine. J Conserv Dent.

[24] Lardani L, Derchi G, Marchio V, Carli E. One-year clinical performance of Activa Bioactive-Restorative Composite in primary molars. Child (Basel). 2022;9(3). 10.3390/children9030433. 10.3390/children9030433 PMC8946891 35327805

[25] Al Tuwirqi AA, El Ashiry EA, Alzahrani AY, Bamashmous N, Bakhsh TA (2021). Tomographic evaluation of the Internal Adaptation for recent calcium silicate-based pulp capping materials in primary teeth. Biomed Res Int.

[26] Rajasekharan S, Vercruysse C, Martens L, Verbeeck L. Effect of exposed Surface Area, volume and environmental pH on the Calcium Ion Release of three commercially available Tricalcium Silicate Based Dental cements. Mater (Basel). 2018;11(1). 10.3390/ma11010123. 10.3390/ma11010123 PMC5793621 29342837

[27] Jaber Ansari Z, Ghasemi A, Norozi H, Akbarzade Baghban A, Samiei M (2022). Microhardness of calcium-enriched Mixture Cement and Covering Glass ionomers after different time periods of application. Iran Endod J.

[28] Mitra SB, Lee CY, Bui HT, Tantbirojn D, Rusin RP (2009). Long-term adhesion and mechanism of bonding of a paste-liquid resin-modified glass-ionomer. Dent Mater.

[29] Hashem DF, Foxton R, Manoharan A, Watson TF, Banerjee A (2014). The physical characteristics of resin composite-calcium silicate interface as part of a layered/laminate adhesive restoration. Dent Mater.

[30] Francois P, Remadi A, Le Goff S, Abdel-Gawad S, Attal JP, Dursun E (2021). Flexural properties and dentin adhesion in recently developed self-adhesive bulk-fill materials. J Oral Sci.

[31] Manihani A, Mulay S, Beri L, Shetty R, Gulati S, Dalsania R (2021). Effect of total-etch and self-etch adhesives on the bond strength of composite to glass-ionomer cement/resin-modified glass-ionomer cement in the sandwich technique - A systematic review. Dent Res J (Isfahan).

[32] Falakaloglu S, Yeniceri Ozata M, Plotino G (2023). Micro-shear bond strength of different calcium silicate materials to bulk-fill composite. PeerJ.

[33] Alipour M, Faraji Gavgani L, Ghasemi N (2022). Push-out bond strength of the calcium silicate-based endodontic cements in the presence of blood: a systematic review and meta-analysis of in vitro studies. Clin Exp Dent Res.

